# Successful Treatment of Alopecia Areata Barbae with Platelet-rich Plasma

**DOI:** 10.7759/cureus.7495

**Published:** 2020-04-01

**Authors:** Hanno Pototschnig, Maximilian T Madl

**Affiliations:** 1 Regenerative Medicine, Munich Medical Esthetic, Munich, DEU; 2 Orthopaedics, Privatpraxis Josephsburg, Munich, DEU

**Keywords:** aesthetics, acp, alopecia areata, alopecia areata barbae, autologous conditioned plasma, beard, dermatology, hair loss, platelet-rich plasma, trichology

## Abstract

Alopecia areata (AA) is a frequent autoimmune disorder in which inflammatory cells attack the hair follicles. AA affecting the beard area is well known and is referred to as alopecia areata barbae (AAB) when involvement is limited exclusively to the beard. Currently, no guidelines are established for specific therapeutic approaches for this condition. We present a case of a healthy 30-year-old male suffering from AAB. Three injections of platelet-rich plasma (PRP) with six-week intervals were applied. Stabilization of the condition was noted at the first follow-up (before the second injection), initial minimal hair regrowth was noted at the second follow-up (before the third injection) and robust regrowth at the one-year follow-up. To our knowledge, this case is the first report of a successful treatment of AAB using PRP. PRP represents a new, safe and potentially effective treatment option for AAB. More studies will be necessary to determine the efficacy of this treatment compared to conventional therapy.

## Introduction

With a lifetime prevalence of approximately 2%, alopecia areata (AA) is a frequent autoimmune disorder in which inflammatory cells attack the hair follicles [1]. Several subtypes of AA exist. AA affecting the beard area is well known and is referred to as AA of the beard (BAA) or AA barbae (AAB) when involvement is limited exclusively to the beard. Frequently steroid injection is used as a treatment modality, but currently, no guidelines are established for specific therapeutic approaches for BAA or AAB [2,3]. Furthermore, no randomized controlled trials for the treatment of BAA have been undertaken [4].

## Case presentation

We present a case of a patient with AAB who had robust regrowth with platelet-rich plasma (PRP) injections. A healthy 30-year-old male with AAB presented with the wish of PRP treatment for aesthetic improvement in the area of his beard. Progressing patchy AAB was present for over two years. Scalp, eyelashes, eyebrows and nails were unaffected. Apart from the AAB, the patient did not suffer from any diseases and did not take any medication. Treatment was administered three times at six-week intervals with autologous PRP. PRP has been prepared as shown in Video 1.

**Video 1:** Preparation of PRP. PRP, platelet-rich plasma.

**Figure 1:**
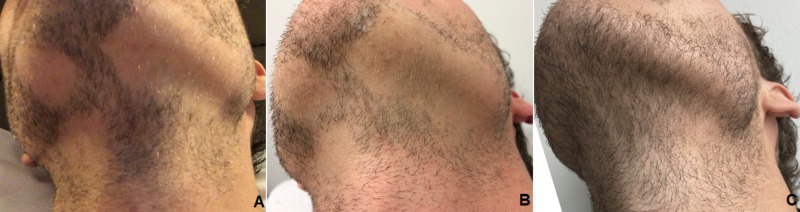
Initial status (A), minimal regrowth six weeks after the second injection (B), robust regrowth at one-year follow-up (C).

For each treatment, 30 ml of blood was taken from the patient via a 21-gauge scalp vein set into two ACP double syringes (Arthrex Inc, Naples, FL). Directly after this, the syringes were centrifuged horizontally for five minutes at approximately 350 G. In each syringe, the PRP settled in the upper third of the syringe (approximately 5 ml), and was drawn into the inner syringe. The inner syringe was twisted out. Following topical anesthesia and disinfection, PRP injections were performed 1 cm apart at a depth of 2-3 mm in serial puncture technique in the affected area (approximately 80 cm²) using 34 gauge needles. Additional methods of platelet activation e.g. addition of thrombin or calcium gluconate were not used in the treatment protocol for this patient. Overall, the procedure was well tolerated. The typical burning sensation during injection was not reported. This could be explained due to the fact that the PRP contained no anticoagulants, which have an unfavorable pH value and could therefore cause a burning sensation. Apart from minimal discomfort within the first 36 hours after injection, no adverse effects were reported. Stabilization of the condition was noted at the first follow-up (before the second injection), minimal hair regrowth was noted at the second follow-up (before the third injection) and robust regrowth at the one-year follow-up (Figure 1).

## Discussion

PRP is considered to initiate wound healing through secretion of various growth factors and cytokines. Antiapoptotic effects on dermal papilla cells and prolongation of the anagen phase through increases in B-catenin and fibroblast growth factor-7 have been shown [5]. Over the last years, PRP became more and more popular for the treatment of hairloss. Especially for androgenetic alopecia a wide body of evidence has recently emerged, demonstrating a positive response from PRP treatments. According to the review from Chen et al., 21 studies reported positive outcomes by objective criteria (88%), while three suggested that there was no clinical improvement, although in two of these studies patients still reported increased satisfaction. There were no complications reported other than transient edema/erythema and pain/headache associated with the procedure. PRP is considered a low-risk intervention to treat androgentic alopecia associated with good patient satisfaction and objective improvements in outcomes [6]. In comparison to androgenetic alopecia, fewer studies have investigated the efficacy of PRP for AA. Donovan reported successful treatment of corticosteroid-resistant ophiasis-type AA with PRP [7]. A recent trial showed that patients treated with topical minoxidil and PRP have both significantly increased hair regrowth compared to placebo and patients treated with PRP have a significantly earlier response than topical minoxidil [8]. In a randomized study, PRP demonstrated significantly improved hair regrowth compared to placebo and triamcinolone scalp injections without any noted adverse events [9]. However, a variable effect was reported in another trial in chronic severe AA [10]. To our knowledge, our case is the first report of a successful treatment of AAB using PRP. PRP represents a new, safe and potentially effective treatment option for AAB. More studies will be necessary to determine the efficacy of this treatment compared to conventional therapy.

## Conclusions

An emerging body of evidence suggests that PRP is a safe and effective treatment modality for androgenetic alopecia. Initial studies report promising results after PRP injections for AA as well. Concerning the subtype of AAB, our case seems to be the first report of a successful treatment. Further studies are needed to confirm these encouraging findings.
